# Lived experience of fully closed-loop insulin delivery in adolescents with type 1 diabetes and HbA1c above target

**DOI:** 10.1089/dia.2025.0062

**Published:** 2025-05-30

**Authors:** Nithya Kadiyala, Janet Allen, Rama Lakshman, Charlotte K Boughton, Malgorzata E Wilinska, Ajay Thankamony, Sara Hartnell, Hood Thabit, Ruben H Willemsen, Pratik Shah, Julia Ware, Roman Hovorka

**Affiliations:** 1 Wellcome-MRC Institute of Metabolic Science-Metabolic Research Laboratories, University of Cambridge, Cambridge, United Kingdom. CB2 0QQ; 2 Department of Paediatrics, University of Cambridge, Cambridge, United Kingdom, CB2 0QQ; 3 Wolfson Diabetes and Endocrine Clinic, Cambridge University Hospitals NHS Foundation Trust, Cambridge, United Kingdom, CB2 0QQ; 4 Diabetes, Endocrinology and Metabolism Centre, Manchester University NHS Foundation Trust, Manchester Academic Health Science Centre, Manchester, United Kingdom. M13 9WL; 5 Division of Diabetes, Endocrinology and Gastroenterology, Faculty of Biology, Medicine and Health, University of Manchester, Manchester, United Kingdom, M13 9PT; 6 Department of Paediatric Diabetes and Endocrinology, The Royal London Children’s Hospital, Barts Health NHS Trust, London, United Kingdom, E1 1BB

**Keywords:** Type 1 diabetes, fully closed-loop, adolescents, automated insulin delivery, artificial pancreas

## Abstract

**Aims:**

The aim of this qualitative study was to explore the impact of using the CamAPS HX fully closed-loop system, which does not require carbohydrate counting, meal announcements or premeal bolusing, on the daily lives of adolescents with type 1 diabetes and HbA1c above the recommended target (≥ 7.5% [58mmol/mol]).

**Methods:**

Twelve adolescents took part in virtual semi-structured interviews. Data was analyzed thematically using an inductive-deductive approach. Study participants also completed quality of life questionnaires.

**Results:**

All interviewees reported reduced effort in managing diabetes, as they no longer needed to count carbohydrates or bolus, and worried less about their glucose levels. This led to improved quality of life, with a greater sense of freedom and normalcy, particularly around meals. A few also noted benefits in physical activity, sleep, work and social life. Interviewees expressed dissatisfaction with the algorithm’s slow response to postprandial glucose spikes, and the need for a tethered pump. Questionnaires showed no significant differences in hypoglycaemia fear or diabetes distress between study periods but reflected a positive experience with the closed-loop system.

**Conclusions:**

In adolescents with type 1 diabetes, fully closed-loop insulin delivery reduced the daily burden of self-management, leading to improved quality of life.

Clinical trial registration: NCT05653050

## Introduction

Adolescence is a challenging period and individuals with type 1 diabetes often experience sub-optimal glucose outcomes and high levels of diabetes distress [1]. Registry data from the United States and northern Europe indicate that HbA1c levels increase during adolescence and remain high into early adulthood [2].

While hybrid closed-loop systems have shown glycaemic and quality of life benefits for adolescents [3], some management burden persists as all commercially available systems require pre-meal boluses or meal announcements for optimal efficacy [4]. Carbohydrate counting is arduous, error-prone and requires a certain level of numeracy and health literacy. Additionally, adolescents tend to be less likely to engage with the hybrid closed-loop system and thus more likely to miss insulin boluses than other age groups [5–7].

Fully closed-loop insulin delivery eliminates the need for carbohydrate counting, meal announcements and pre-meal bolusing. In the CLEAR Phase 2 randomized controlled trial, adolescents with type 1 diabetes and sub-optimal HbA1c showed improved time in range using the fully closed-loop CamAPS HX system compared to non-automated pump therapy with sensor (45% vs 32%, mean difference 12.9 percentage points) [8]. In the present psychosocial sub-study, we used semi-structured interviews and validated questionnaires to examine the impact of using the fully closed-loop system on adolescents with type 1 diabetes.

## Methods

### Overview

**Figure 1:**
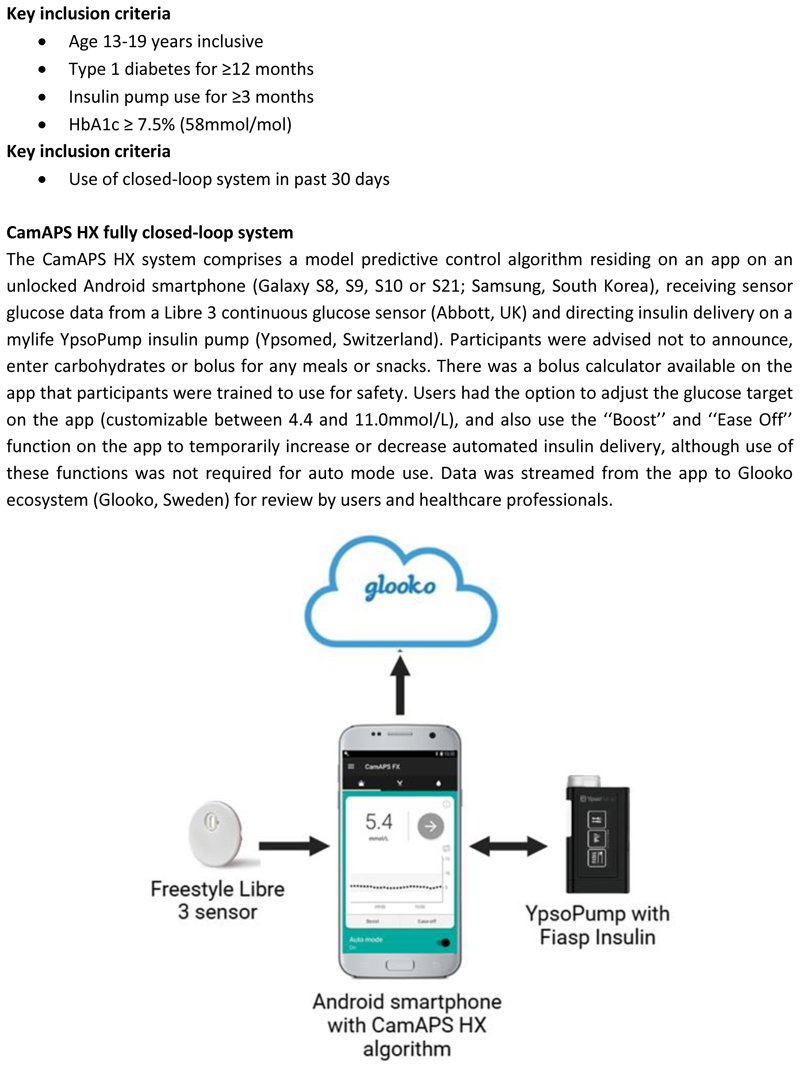
Key trial eligibility criteria and description of the CamAPS HX fully closed-loop system.

Participants completed questionnaires as part of the CLEAR Phase 2 study and were invited to participate in interviews [8]. This was a two-centre randomised crossover trial involving 24 adolescents (aged 13-19 years) with type 1 diabetes and HbA1c above recommended targets (≥7.5% [58 mmol/mol]) using non-automated insulin pump therapy at baseline[8]. Participants were randomised to eight-weeks use of a fully closed-loop system (CamAPS HX; CamDiab, UK) with faster-acting insulin aspart (Fiasp; Novo Nordisk, Denmark) or eight-weeks use of their usual insulin pump therapy with Libre 3 continuous glucose sensor (Abbott Diabetes Care, UK), before crossing over to the other arm. The CamAPS HX fully closed-loop system receives continuous glucose monitoring data from the FreeStyle Libre 3 sensor and uses a model predictive control algorithm to direct insulin delivery on the mylife YpsoPump insulin pump (Ypsomed, Switzerland). It does not require carbohydrate counting, pre-meal bolusing or meal announcements. Insulin sensitivity and active insulin time are calculated automatically. The system includes optional functions of “Boost” and “Ease Off” that allow users to increase or decrease the intensity of algorithm-driven insulin delivery as needed. Their use was entirely optional and participants were instructed not to use them routinely. Further details about the study are provided in Figure 1.

### Recruitment and data collection

All participants completed three validated questionnaires at baseline to assess hypoglycaemia fear (Hypoglycaemia Fear Survey-Child [HFS-C]), diabetes distress (Problem Areas in Diabetes Teen [PAID-T]) and expectations of how the system could improve their diabetes-specific well-being (INsulin Dosing Systems: Perceptions, Ideas, Reflections, and Expectations [INSPIRE for youth]) [9–11]. Participants completed the HFS-C and PAID-T at the end of each study period. The INSPIRE for youth questionnaire and additionally the Closed-Loop Experience Questionnaire were completed at the end of the fully closed-loop period (FCL) only.

On finishing the study, participants were invited, face-to-face and via email, by healthcare professionals involved in their clinical care to take part in a virtual interview exploring their experiences using the fully closed-loop system. Purposive sampling was used to ensure diversity in age, gender and ethnicity. Participants aged 16 years and over, and parents and guardians of participants under 16 years provided informed consent. Written assent was obtained from participants under 16 years. Recruitment and data collection continued until thematic data saturation was reached.

Interviews were audio-recorded, using Zoom or Microsoft Teams. Participants were interviewed once at the end of the study, and their parents were given the option of being present. Interviews were conducted by NK, a female clinical researcher trained in qualitative research methods. NK was involved in training two of the twelve interviewees on the closed-loop system.

We used a semi-structured interview design based on a topic guide (Supplementary Table 1) to ensure key study aims were addressed while allowing participants flexibility to discuss issues they felt were important. The topic guide was informed by a literature review of user experiences of closed-loop systems and consultation with members of the clinical team, and was revised to based on emergent findings.

The interviewer was involved in the clinical care of two of the twelve interviewees. To mitigate potential bias, we followed a structured topic guide to ensure consistency across interviews, emphasised to interviewees that both positive and negative responses were valued, and maintained a neutral interviewing stance. In addition, the interviewer kept an ongoing memo paying attention to negative points mentioned by interviewees, held regular reflexive team discussions and had colleagues review coding frameworks.

### Ethics

Ethics approval for the CLEAR study and interview sub-study was received from the independent East of England – Cambridge Central Research Ethics Committee (UK) prior to study commencement.

### Data analysis

Interview transcripts were anonymized and analyzed thematically using an inductive-deductive approach. Data collection and analysis were done concurrently to ensure findings identified in early interviews could inform the topics explored in later accounts. Interview transcripts were analyzed and compared to identify recurring themes. Three additional members of the research team reviewed the coded datasets to ensure the final coding framework reflected the key themes. Our reporting of methods and findings was guided by the Consolidated Criteria for Reporting Qualitative Studies (COREQ) [12]. The qualitative analysis was facilitated using NVivo 14 (QSR International, Australia). Questionnaire data analyses were carried out using SPSS Statistics software, version 29 (IBM Software, UK).

## Results

**Figure 2:**
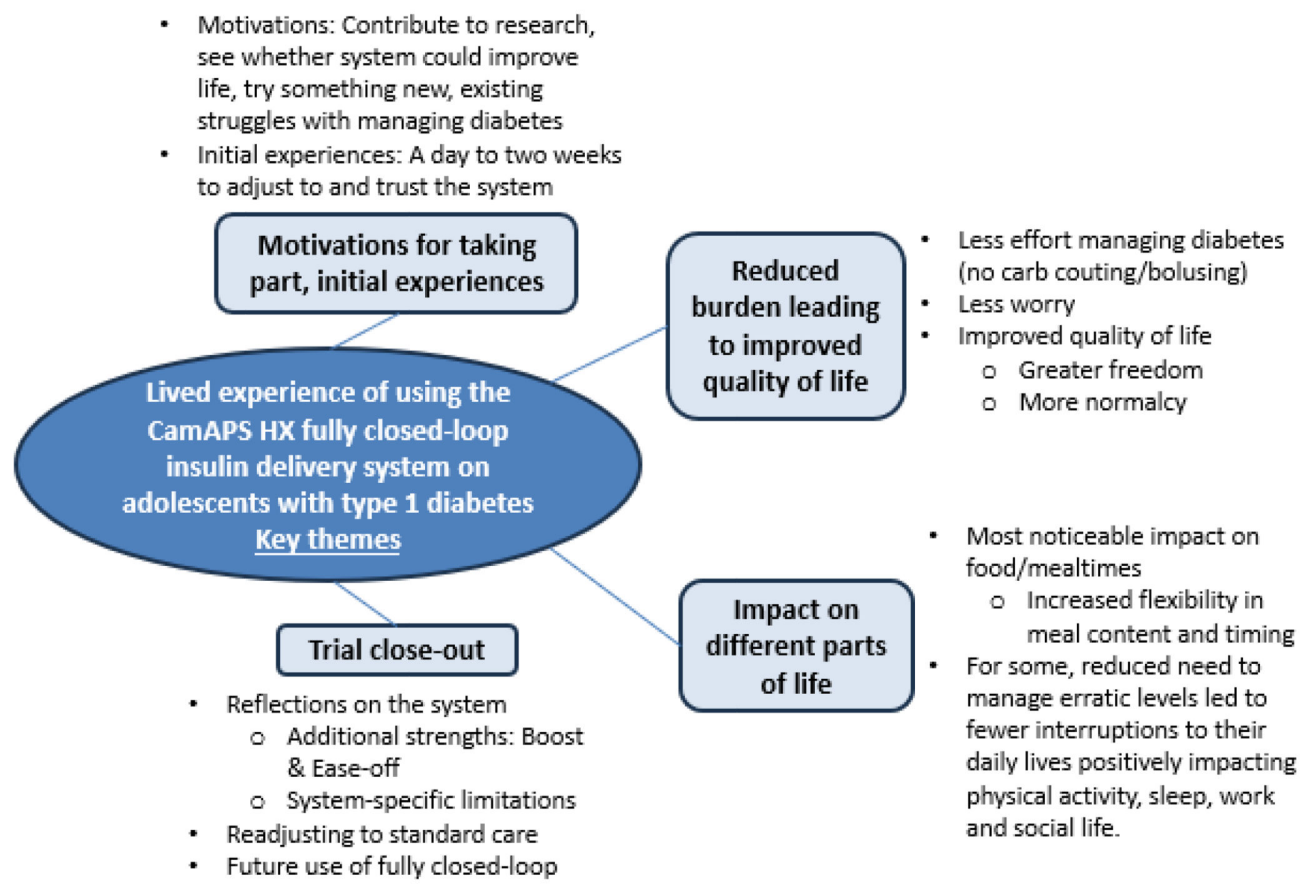
Themes and sub-themes of adolescents’ experience of using the CamAPS HX fully closed-loop system.

Between November 2023 and September 2024, twelve participants were interviewed. Each interview lasted approximately forty minutes. Demographic data are presented in Table 1. The results are structured according to the key themes and sub-themes (Figure 2). Demographic details are provided after each participant quotation. Unique identifiers for each interviewee (i.e., 01-12), their age and gender (M=Male, F=female) are used for anonymity. Further participant quotations for each theme are provided in the supplementary material (Supplementary Tables 2-5).

### Motivations for taking part and initial experiences

#### Motivations for taking part in the trial

Adolescents’ motivations for taking part included a desire to contribute to research: “It helps find new ways to help diabetics, not just me” (10_18y_M); curiosity about whether the system could make their “life even easier” (04_20y_F), an interest in trying something new and existing struggles with managing diabetes: “I was doing pretty badly. I used to sneak food without putting it in my blood” (i.e. eat without bolusing) (02_14y_F).

#### Initial experiences including adjustment period and trust

Nearly all interviewees needed between a day to a couple of weeks to adjust to wearing the system. At first, they described how it was “weird” (01_20y_M) not having to bolus, and it took time to develop trust in the system and its ability to regulate glucose levels: “It was just me trying to fully understand and get into a routine basically and knowing that the system is accurate” (07_17y_M). However, several interviewees enjoyed using it “within the first day” (01_20y_M), indicating it was “quite easy to get used to” (03_15y_M) and quite a few adolescents trusted it “straight away” (09_13y_F). “Oh, it was amazing. I loved it cause the minute I came on to it, I was screaming to my mum saying Oh my God, I’ve don’t have to carb count anymore” (02_14y_F).

### Reduced burden leading to improved quality of life

Adolescents highlighted the reduced effort required to manage their diabetes including the convenience of “not having to carb count” (05_13y_F) or bolus:

“It was easier to manage my blood sugar. When my sugar’s high, I don’t need to do correction or when I eat, I don’t need to do anything… I don’t get burnt out by putting insulin stuff every time” (11_14y_M).

They described feeling less worried about their glucose levels due to the system’s ability to regulate them:

“This study has made my life so much more easier… It really helps the person a lot. It removes the burden of having to care so much… Yeah, I wouldn’t have to worry that much. Most of the time, I wouldn’t even think about it ‘cause I knew the system could handle it… It would just bolus by itself… There was never oh, I have to check my blood sugar. Did I bolus or did I not? It will just relieve that from my mind” (12_15y_M).

This reduced burden contributed to an improved quality of life, with interviewees expressing a greater sense of freedom and normalcy:

“I would say in a way it is a manageable cure to diabetes… Within the first week, I basically turned to my friends and saying, well, not going to lie to you guys, I’m not really diabetic at the moment. I don’t have to do anything… For the majority of the time, I was acting and behaving like everyone else. I guess I could constantly stay zoned out. I never had to zone back into controlling my sugars unless I had to do a set change” (01_20y_M).

A couple of interviewees also reported a positive impact on their mood:

“Before the trial, the diabetes was affecting a lot of things. It was definitely affecting energy levels and my mood… Yeah, it completely changed. I felt like I could get up, do whatever I needed to do, go out more. My mood’s certainly improved as well. I think it was just because I didn’t need to worry so much about it” (10_18y_M).

### Impact on different parts of life

#### Most noticeable impact on food/mealtimes

In general, interviewees reported having similar meals during both study periods but use of the fully closed-loop system afforded them greater flexibility in meal content and timing: “I could eat whatever I want and the insulin would do itself” (03_15y_M). This was because of not having to count carbohydrates, bolus or check their glucose levels as often. A few spoke of how it was “easier” (09_13y_F) to manage meals out.

Some adolescents took advantage of the system to increase consumption of less healthy foods but showed awareness of the potential adverse health impacts. On the other hand, some interviewees made more “cautious” (02_14y_F) food choices due to the study pump’s small insulin cartridge and algorithm’s slowness to responding to high postprandial glucose levels.

#### Impact on sleep, physical activity, social life, school and work

Although most interviewees reported no change, some, particularly the older adolescents, highlighted how they saw improvements in other aspects of their life too.

They noted being more active and fewer interruptions to sport due to the closed-loop’s ability to maintain glucose levels. Some reported improved sleep due to fewer out of range glucose levels overnight. “There wasn’t much restless sleep. A knock-on effect of I know my sugars will control themselves overnight. I don’t have to stay up to control it.” (01_20y_M). Fewer interruptions due to diabetes also meant interviewees saw improvements in their “focus” (04_20y_F) and work.

For some, the fully closed-loop enhanced their social life. A couple of interviewees reported going out was “easier” (06_18y_M) due to less worry over hypoglycaemia. Two interviewees appreciated not needing to use their phone or pump to bolus when out with peers or at school saving embarassment. Some reported feeling more present in their daily lives and interactions with loved ones:

“I didn’t have to focus on my diabetes as much… I was able to spend more time with friends and family and doing the things I enjoy without worrying about my diabetes as much” (10_18y_M).

### Trial close-out

#### Additional strengths of the system

Interviewees found the options of Boost and Ease-off “useful” (02_14y_F) and “effective” (04_20y_F). They reported using Boost more frequently than Ease-off, typically for thirty minutes to an hour, two to three times a week. Boost was particularly helpful when “blood levels would go high for a long amount of time” (06_18y_M), such as when they’d eaten “something sugary or high in carbs” (12_15y_M). Those who used ease-off did so primarily to prevent hypoglycaemia with physical activity.

#### Limitations of the system

Three interviewees felt the system was too slow in responding to high glucose levels: “the highs would end up for most of the afternoon rather than an hour” (04_20y_F). A couple were frustrated at being unable to “switch off the urgent low alarm” (04_20y_F). Several were also inconvenienced by the pump hardware. While some interviewees appreciated the study insulin pump’s small size, many found the insulin cartridge capacity too limited, requiring frequent replacement. Around half of the interviewees also disliked the tubed pump reporting that it often kept getting caught or falling off.

#### Readjusting to standard insulin pump therapy

Adolescents mentioned needing a few days up to a couple of weeks to readjust, often experiencing more erratic glucose levels during this period due to forgetting to bolus. Interviewees at university highlighted the difficulty of restarting usual care combined with work stressors. However, for almost half of the interviewees, this transition was mitigated by being upgraded to hybrid closed-loop therapy following the trial, as part of the national rollout under the NICE technology appraisal TA943 [13].

#### Future use of fully closed-loop

Most interviewees really liked using the fully closed-loop system with some expressing eagerness for future use: “I am hoping that that machine is available to everyone one day” (12_15y_M). Adolescents believed the system would be most beneficial for younger children, individuals who are newly diagnosed, those with difficulty controlling their blood glucose levels or those experiencing stressful life circumstances:

“I think it benefits those who struggle coping with diabetes. It’ll put their mind at ease just knowing that they’ve got something that can help them in the long term.” (10_18y_M)

### Questionnaire results

There were no significant differences between periods in hypoglycaemia fear (FCL: 56 vs usual care: 60, p=0.22) or diabetes distress (FCL: 63 vs usual care: 72, p=0.12) (Table 2). INSPIRE questionnaire scores at baseline (81) and after the fully closed-loop period (70) were generally high (on a 0–100 scale), indicating that respondents had positive expectations and experiences of the system.

Table 3 presents the findings from the Closed-loop Experience Questionnaire. 82% of respondents were happy to have their glucose levels controlled automatically by the system and 95% would recommend it to others. The questionnaire’s free text responses (Supplementary Table 6) echoed themes from the interviews, such as reduced effort with managing diabetes as a result of not having to bolus or carbohydrate count, as well as less overall worry. Reported limitations included the perceived suboptimal response of the algorithm to their glucose levels, particularly when high (27% of respondents), disruptive alarms, and issues with pump tubing.

## Discussion

Our qualitative evaluation of adolescents using a fully closed-loop insulin delivery system for eight weeks without the need for carbohydrate counting, meal announcements or pre-meal bolusing found it reduced the effort and burden of diabetes management, providing greater freedom, normalcy, and an improved quality of life. The benefits were most notable around mealtimes but, for the older adolescents, also extended to physical activity, sleep, productivity and social life.

Participants highlighted the main advantage of the fully closed-loop system as not needing to count carbohydrates or bolus for meals- key requirements for most commercially available hybrid closed-loop systems. Even the iLet system still relies on adolescents to make qualitative meal announcements and initiating pre-meal boluses [14]. This adds to the burden of diabetes management, particularly for adolescents, who often struggle with these tasks.

Another key benefit of the fully closed-loop system was that participants were less worried about their glucose levels, which they perceived to be more stable. This is consistent with the trial findings showing that fully closed-loop use with Fiasp increased time in target range (3.9–10.0 mmol/L) by 12.9 percentage points compared to standard pump with sensor (45% vs. 32%) [8]. Importantly the observed improvement in time in range was achieved while simultaneously reducing burden and improving quality of life.

Our study builds on findings from fully closed-loop use in adults with T1D, who similarly reported reduced burden leading to greater flexibility around food, improved sleep and productivity [15].

These positive factors have also been observed with hybrid closed-loop systems [1,16–18]. However, for adolescents with sub-optimal glycaemic outcomes despite bolusing (over 50% of total insulin was delivered as a bolus in the usual care group in the main study), not having to carbohydrate count or bolus further enhanced quality of life. Some interviewees described feeling freer to engage in activities similar to their peers without diabetes, avoiding constant concerns about glucose levels, which remained more stable. In qualitative studies, adolescents using hybrid closed-loop systems reported disconnecting their devices in public to not stand out [1]. By reducing the need to use their phone or pump for boluses or corrections, the fully closed-loop system additionally offered discretion and reduced visibility of diabetes in social and academic settings. These are important considerations when evaluating the acceptability and impact of diabetes technologies in youth, in whom diabetes self-management is complicated by physiological endocrine changes associated with puberty, fear of hypoglycaemia, a desire to socialize and fit in with peers and self-consciousness around their diabetes [1,5,19]. Despite the many benefits of fully closed-loop therapy in this high-risk cohort, some adolescents reported unhealthier eating behaviours due to the increased flexibility provided by closed-loop. Similar findings have been observed in hybrid closed-loop studies and our fully closed-loop study in adults [15,20]. Effective implementation of this technology should consider nutritional and behavioural education to promote healthy eating habits [20].

Limitations in system use were often related to hardware challenges, such as the frequent need to replace the insulin cartridge and issues with pump tubing. The latter might have been particularly challenging for those using the Omnipod patch pump during usual care (50% of interviewees). These system-specific features are key considerations for healthcare professionals when tailoring devices to individual preferences. Around 30% of interviewees and questionnaire respondents were dissatisfied with the algorithm’s responses to their glucose levels particularly when high. The slow postprandial response is a commonly reported limitation of closed-loop systems using currently available subcutaneous rapid-acting insulins [14,21].

Participants had high expectations of the fully closed-loop system as reflected by the high INSPIRE score at baseline. Given the system is limited by subcutaneous insulin absorption and uses hardware that may be different to participant’s regular management, healthcare providers should discuss realistic expectations with new users prior to initiation and regularly assess participant’s experiences of the system to optimize use [4]. No significant improvements were observed in questionnaire diabetes distress and hypoglycaemia fear scores. These questionnaires, which measure broad dimensions of distress rather than closed-loop specific metrics, may require longer-term follow-up and a larger sample size to detect a difference [22]. Other psychosocial evaluations of hybrid closed-loop systems in adolescents using questionnaires have also yielded inconsistent results, despite positive interview responses and improvement in glycaemic outcomes [23,24].

The algorithm may not be sufficiently adaptive to meet the needs of all adolescents with type 1 diabetes and indeed some may prefer to have more opportunities to engage with the system to fine-tune their blood glucose levels. However, as suggested by interviewees themselves, the fully closed-loop system could be beneficial for those struggling with blood glucose control, experiencing diabetes distress or stressful life circumstances. Higher levels of distress are significantly associated with poorer self-management behaviours and worse clinical outcomes [25]. Adolescents navigating stressful life events such as moving out of the family home, starting university or a new workplace, may have reduced mental and cognitive reserve to consistently manage their diabetes effectively. For these individuals, a fully closed-loop system could offer meaningful support, even if used temporarily as a form of respite.

A key strength of our study was the use of semi-structured interviews as well as questionnaires, enabling in-depth exploration of adolescents’ views on the use of a novel system. Interviews may allows us to understand the ideas most important and meaningful to participants, which may not captured by questionnaire data alone [24]. Participants were from diverse ethnic and socio-economic backgrounds. Limitations include the small sample size, inclusion of a broad age range from early adolescents to young adults, who may have different experiences of their diabetes management, involvement of insulin pump users only, who may have found it easier to adapt to the new system, and the close support participants received as part of the research trial potentially limiting the generalizability of our findings. The interviewer was involved in the clinical care of two of the twelve interviewees, which could have introduced bias and encouraged interviewees to report more positive experiences, but steps were taken to mitigate this as explained in the Methods. Longer-term follow-up is needed to assess whether the benefits of fully closed-loop reported in the interviews are sustained over time and to explore its broader impact on self-management behaviours and participants’ overall relationship with their diabetes.

## Conclusions

In adolescents with type 1 diabetes, fully closed-loop insulin delivery, which does not require carbohydrate counting, meal announcements or pre-meal bolusing, reduced the daily burden of managing diabetes, leading to improved quality of life. The system shows promise in addressing the high levels of diabetes distress and sub-optimal glucose outcomes often seen during the challenging transition from childhood to adulthood. Healthcare professionals and policymakers must prioritize psychosocial factors alongside glycaemic outcomes to realize the full potential of diabetes technology in providing effective, holistic care for individuals with type 1 diabetes.

## Supplementary Material

Supplementary tables

## Figures and Tables

**Table 1:** Demographic characteristics of participants taking part in interviews (n = 12).

Characteristic	
Age at time of interview (years)	16.4±2.5 (range 13.6-20.7)
Gender – Female *n (%)*	5 (42)
Race/ethnicity *n (%)*	
Caucasian	7 (58)
South Asian	3 (25)
Black African	1 (8)
More than one race	1 (8)
Parents’ highest degree *n (%)*	
University	3 (25)
College/A levels/equivalent	3 (25)
School/GCSEs/equivalent	2 (17)
No GCSEs	1 (8)
Missing data	3 (25)
Mean duration of diabetes (years)	9.6±5.4 (range 3.0-17.7)
Education and living situation	
School (living at family home)	9 (75)
University (living at family home)	1 (8)
University (living independently)	2 (17)
Type of pump used prior to trial *n (%)*	
Medtronic 640G	2 (17)
Medtronic 670G	2 (17)
Medtronic 780G	1 (8)
Omnipod	6 (50)
YpsoPump	1 (8)
Type of CGM used prior to trial *n (%)*	
Dexcom G6	4 (33)
Dexcom G7	2 (17)
Libre 2	5 (42)
Libre 3	1 (8)
HbA1c at baseline (%)	9.2 (8.3, 9.9) (range 7.5-13.5)
HbA1c at baseline (mmol/mol)	78 (67, 85) (range 58-124)

**Table 2:** Questionnaire results

	Baseline	Fully closed- loop	Usual care (standard pump with CGM)	P value^a^
Hypoglycaemia Fear Survey- Child^b^	(n=24) 59.3 ± 15.3	(n=21) 56.2 ± 15.2	(n=23) 59.7 ± 12.1	0.22
Problem Areas in Diabetes Teen^c^	(n=23) 71.2 ± 25.7	(n=21) 63.4 ± 29.7	(n=22) 71.9 ± 28.9	0.12
INSPIRE foryouth^d^	(n= 24) 80.7 ± 12.4	(n=22) 69.6 ± 17.9	-	-

Data are presented as mean±SD for normally distributed values.

a Based on a linear mixed model, adjusting for period as a fixed effect and accounting for the baseline value as a separate period.

b A lower score reflects less fear of hypoglycaemia and, consequently, a more favourable outcome.

c A lower score indicates fewer problems or less distress related to diabetes management and, consequently, a more favourable outcome.

d A lower score at baseline reflects lower positive expectations that an automated insulin delivery (AID) system can improve overall diabetes-specific well-being, and consequently, a less favourable outcome. A lower score after AID use reflects less favourable experience with the AID.

**Table 3:** Results from the Closed-loop Experience Questionnaire (n=22).

	Strongly agree	Agree	Neutral	Disagree	Strongly disagree
Q1. I was happy to have my glucose levels controlled automatically by the system	14(64)	4 (18)	4 (18)	0 (0)	0 (0)
Q2. I spent less time managing my diabetes (glucose testing, adjusting insulin therapy, keeping a diary, data review...)	13 (59)	7 (32)	2 (9)	0 (0)	0 (0)
Q3. Using the system took more time and work than it is worth	0 (0)	1 (5)	3 (14)	10 (45)	8 (36)
Q4. I was less worried about my glucose control	11 (50)	7 (32)	4 (18)	0(0)	0 (0)
Q5. I slept better during the nights	10 (45)	2 (9)	7 (32)	1 (5)	2 (9)
Q6. I would recommend closed-loop to others	15(68)	6 (27)	1 (5)	0 (0)	0 (0)

*Values are n (%)*

## Abbreviations

FCL: Fully closed-loop period
HFS-C: Hypoglycaemia Fear Survey-Child
INSPIRE: INsulin Dosing Systems: Perceptions, Ideas, Reflections and Expectations
PAID-T: Problem Areas in Diabetes Teen
