# Supplementary material for: Lived experience of fully closed-loop insulin delivery in adolescents with type 1 diabetes and HbA1c above target
Source: Diabetes Technol Ther. Author manuscript; Available in PMC 2026 Jan 29. (PMC7618673; doi:10.1089/dia.2025.0062)
Supplement: Supplementary tables [file EMS211981-supplement-Supplementary_tables.pdf]

Contents

Supplementary Table 1. Topic guide. 2

Supplementary Table 2. Theme: Motivations for taking part and initial experiences. 3

Supplementary Table 3. Theme: Reduced burden of diabetes management leading to improved quality of life. 4

Supplementary Table 4. Theme: Impact on different parts of life. 7

Supplementary Table 5. Theme: Trial close-out. 10

Supplementary Table 6. Free-text results from the Closed-loop Experience Questionnaire. 14

Supplementary Table 1. Topic guide.

- Previous experiences of diabetes technology
- Motivations for taking part in the trial and initial expectations
- Ease of use of study equipment
    - Training visit
    - Boost and Ease-off use
- Initial experiences of using the fully automated system, including adjusting to not announcing meals / bolusing
- If, and when confidence and trust in the system was established
- Impact of using the fully automated system on:
    - Food and mealtimes
    - Physical activity
    - Sleep
    - Social and family life
        - Impact on loved ones
        - Alcohol
    - School and work
- Impact of system on the practical and mental burden of diabetes management
- Views on trial close-out, and going back to standard insulin pump therapy
- Views about how the system could be improved to aid efficacy and acceptability
- Views on which individuals would most benefit from using a fully closed-loop system

Supplementary Table 2. Theme: Motivations for taking part and initial experiences.

Sub-theme	Participant quotations
Motivations for taking part in the trial	I would like to help, not necessarily try find a cure, because it's not just me, it's much more towards the research part. But if I can help give feedback and development towards trying to make it easier for everyone else, then I'd like to do that… if I'm able to say, hey, this does work, this doesn't work, then I feel like I've contributed in some way (01_20y_M).<br><br>Really curious as to like. How it could help me and benefit me in the future (07_17y_M).<br><br>Because it's just like an experience. Something for you to try out (08_14y_F).<br><br>I've had difficulty like controlling my blood glucose levels so they don't jump up and down (10_18y_M).
Initial experiences including adjustment period and trust	<u>Adjusting to wearing the system</u><br>(Regarding use of the study insulin pump:) It was different. It took me a few days to try fully get through it and a few messages back and forth with *research nurse* and the rest of the team, but I think at the end of the day, it's very similar… It's got the same sort of idea behind it as well. So, I wouldn't say it was too difficult, but it's just different. It's just you having to relearn how to use the pump again (01_20_M).<br><br>(Regarding the infusion sets): When me and my mum tried to change it by ourselves, it was a bit stressful. We didn't know how to do it properly and it took quite a bit of time on our first attempt. But then as we kept doing it, it just became more easier. Yeah, we use the user manual quite a lot (12_15y_M).<br><br><u>Not having to bolus</u><br>It was quite strange at first not to have to do anything, but it was really nice. I think I probably watched the app a lot more in the beginning to see kind of what was going on with it (04_20y_F).<br><br>Cause at the back of my mind I always thought I needed to do insulin, but then I realized that it's doing it for me (10_18y_M).<br><br><u>Establishing trust in the system</u><br>The first time I was a little bit scared, but then we realized there wasn't (anything bad) actually gonna happen. So I just stopped being scared (05_13y_F).

	The first week I didn't know if it was gonna be that good… If my blood sugar's probably gonna go high much more. But then, after the first week I saw it was actually doing a good job. So I was very confident in it (12_15y_M).<br><br>Probably like a week or two down the line. My levels are like starting to become more stable, so give and take… (It took this long:) Probably because like I said, it was just a new pump overall and my level was still running high during the 1st 2-3 days. But then after I'd say it started to go lower and lower became more stable (03_15y_M).<br><br>At first I was I was a bit unsure about it, but once I'd gotten used to it, I had no thoughts about it after… I had constant reassurance from my parents and *research nurse* (research nurse) saying it was going to be alright, that I could do it… Also knowing that *research nurse* was only a phone call away or someone was a phone call away to help if I needed it (10_18y_M).<br><br><u>Positive initial experience</u><br>I thought it was good. Yeah, 'cause I thought it would be quite overwhelming, like managing it, but it was good (07_17y_M).<br><br>It was like, really easy to use and useful and I just really liked it, basically (09_13y_F).<br><br>I think I trusted it quite well from the start. Because I know how much work has gone into it and like the technology is very good (04_20y_F).

Supplementary Table 3. Theme: Reduced burden of diabetes management leading to improved quality of life.

Sub-theme	Participant quotations
Less effort to manage diabetes due to not having to carbohydrate count or bolus	I found it much easier. Because the pump would probably inject insulin automatically without me having to worry about it (03_15y_M).<br><br>I think that was my main part. Not having to bolus, it was just very convenient (06_18y_M).<br><br>I didn't have to think about carb counting. I could just pretty much eat as long as I was careful about my blood sugar after I'd eaten (07_17y_M).<br><br>We'd normally have to sort of weigh out her food and. Especially things like mash, but I didn't have to worry about doing that. I just dished up a portion and then obviously not ridiculous amounts. But yeah, she just come down and eat normally without having to do her insulin beforehand (parent of 08_14y_F).

More stable glucose levels and less worry	<u>More stable glucose levels</u><br>I guess it's like improved quite a bit… My rates and everything aren't perfect because of it, but they've gotten from like a very bad situation to like a you could do better, but it's not that bad anymore situation (06_18y_M).<br><br>Yeah, there's a lot of improvement. And as I said before, they were a lot more in range. I had less highs and lows (10_18y_M).<br><br>The hypos and hypers kind of died down a lot (01_20y_M).<br><br>It definitely improved my blood sugars and all of that. After a few days it started like I was in like target quite a lot (09_13y_F).<br><br>I didn't think it was real, but then I was shocked how it actually worked. It would actually put in the correct amount of insulin most of the time (12_15y_M).<br><br><u>Less worry</u><br>I just went out more. It was a much happier time of life. I guess there was less worry. You didn't have to think about it. You weren't consciously worrying about it throughout the day (01_20y_M).<br><br>I guess my mental burden decreased a lot because I didn't have to worry or check my blood level. Or I checked my blood level at this anyways, just in case, but I don't have to worry over it being messed up or too high at random parts or times of the day. So it was quite helpful… It was very convenient. I didn't really have to worry or think about it all the time, and I could just check once in a while to be like, oh, it's doing well. It's doing fine. So it was very convenient (06_18y_M).<br><br>The fact that it just altered my blood sugar, I found that really useful. Knowing that I barely did anything apart from the boost functions… I was quite happy with the result 'cause it was just the system was quite impressive (07_17y_M).<br><br>I'm spending more time worrying about my blood sugars (with injections). When I was doing injections, but with the closed-loop I felt comfortable knowing that I didn't need to put as much effort into managing them (10_18y_M).
Greater freedom	Yeah, like I felt free. It was a lot easier and I could do what I wanted (05_13y_F).<br><br>I loved it. I literally loved it, so I didn't have to do, you know, do any carb counting. I could just do what I want (02_14y_F).<br><br>I was happily going out. I was playing football whenever I wanted to, I was eating whenever I wanted to (01_20y_M).

More normalcy	I felt like kind of normal again before I had diabetes. You know, since like it was much easier eating without having to carb count (02_14y_F).<br><br>Just like the uninterrupted and not having to constantly think about something and come back to it and always have it in the back of your mind. It just means you can focus on what you're doing a lot more and. Put more energy into that, I suppose (04_20y_F).
Improved mood	People were saying like well your mood's definitely improved. Not saying that I'm a miserable person, but when my sugars do start going up and down, it can get very difficult to try still behave normally or still treat everyone and speak to everyone normally. That had kind of died down quite a lot. It was nice… But, yeah, it was less mood swings, more control. Your body's not continuously tired from going up and down triggers, and you feel like you're able to give everything 100% all of a sudden, which, like I was saying, is weird because it's kind of one of those well, is this what not feeling diabetic feels like for everyone continuously, instead of, like, the mood swings every once in a while of feeling like your body's drained out of no reason? (01_20y_M).

Supplementary Table 4. Theme: Impact on different parts of life.

Sub-theme	Participant quotations
Food	<u>Flexibility in meal content and timing</u><br>I don't think it really impacted the choices, but I think it helped me worry less about them, like I would still have the same things I wanted to eat, but I didn't have to kind of think about it as much or worry about the impact that was gonna have (04_20y_F).<br><br>I could eat whenever I wanted to, without needing to worry about my blood sugars going through the roof. When I wasn't on the closed-loop system, I was worrying about how many carbs are in certain foods? How much do you put in insulin wise? Whereas with the closed-loop system, I didn’t worry at all (10_18y_M).<br><br><u>Managing meals out</u><br>And like going out to eat for meals and stuff you don't have to do anything different (04_20y_F).<br><br>Actually I preferred it as I didn't have to like bring out my phone and my Omnipod to add all the carbs. Like easier to like do (09_13y_F).<br><br><u>Took advantage of the system to indulge</u><br>But then, most of the time I would get maybe ahead of myself and think, oh, I can just eat whatever I want. I don't need to bolus. Make sure I took advantage of it… I would have more unhealthy stuff, and sometimes I wouldn't tell my mom about it because I'll just Boost and then the sugar will go back to normal (12_15y_M).<br><br>“I put the system through its test, I guess fully went for it… There was an all you can eat buffet for my friend's 21st birthday… There were many pizzas, a lot of hard carbs, basically. So it (the system) was run through its paces, but it dealt with it quite well… Yeah, I was still kind of working out, still make sure I was keeping fit so I didn't just suddenly shoot my weight up. And then I've got different problems then.” (01_20_M).<br><br>Towards the end, we kind of got off track. We was just like, you know what, let's just go out because, you know, it's like your final week on this pump. Then you're going to have to go carb counting. So let's just have a celebration. And I could say I spent well over like 600 carbs that night. Cakes, donuts, ice cream… You know, you can eat whatever you want and you can feel like how you was before you was diagnosed… But don't take too much of advantage of it. You know, you don't want to be eating all these fatty foods. Like too much and everything. (02_14y_F).

	<u>More cautious with food choices</u><br>And given that the insulin also runs out quickly in that pump so I was a bit wary about what food I was eating. I would probably say like the first like 2 weeks… I wanted to make sure you know I wasn't giving too much carbs or too much insulin would run out. But I would say I was eating similar stuff, but at the same time I was trying to watch what I eat (02_14y_F).<br><br>I guess I tried to decrease how much I had at one time or instead of having like little bits for a large amount of time, I had it like in one meal and then I wouldn't eat for like the rest of the day. Or I'd eat it a lot longer later. Just so I could like at least let my blood level go back to normal levels. If anything it helped me because I shouldn't have had that many snacks. Anyways it's more helpful than not (06_18y_M).
Physical activity	So I'm quite active, I'd like to say. So play football for the uni team and also still doing martial arts. That's kind of the two things that I've been doing for the last decade and a half. And there was a lot less I'm having to stop because my sugars have dropped and I feel like I'm going to pass out. So that kind of stopped happening throughout. Same as when I take my pump off. Okay. The sugars will eventually go high, but there was a lot less of it because of how controlled they were before I took my pump off. So it was good to see in a way, is that. I don't know if it sounds cringe, but my performance improved throughout that period (01_20_M).<br><br>Definitely worried less about it because I used to have to beforehand think, oh, what am I going to eat beforehand? What temporary basal can I do? I'd be taking more breaks in between. I think it was more effective at just letting me, you know, go through it, uninterrupted with anything diabetic (04_20y_F).<br><br>When I knew I could do ease off, it just made me more active (12_15y_M).
Sleep	I could sleep peacefully without my mom. Normally, before using the system, she would have woken up in the middle of the night to because like if she would have woken up then because the sugar would have been high and she put in a bit more carbs to make sure it goes down, but now everyone's asleep (12_15y_M).<br><br>Generally, like improved sleep, I think just because the level was constant because I was quite prone to like going low in the night and I do quite a lot of exercise, so it would be quite a recurrent problem and I'd be trying to set up temporary basis and all sorts to get around it. But I think it did make quite a big difference. The only issue is then if the alarms went off. If I did go low then I'd be kept up, but generally it was a big improvement for sleep… They (the system) kind of prevented them (hypoglycaemia episodes) in the first

	place mainly and so it was quite comforting as well to know that that was another thing I didn't have to worry about (04_20y_F).
School and work	Fewer interruptions<br>It was better, weren't it? 'Cause you didn't come out of lessons as much as you do now to manage it (parent of 08_14y_F). (In the past, she would have to come out of lessons due to alarms) 'Cause it's just like loud and disruptive so I normally go out so it doesn't distract others. Think it didn't have really loud alarms, and they were more in range, so they didn't really go off that much (08_14y_F).<br><br>I was able to focus more, I was able to revise more. I wouldn't find myself being distracted as much… (01_20y_M).<br><br>Yes, but in a positive way. I wouldn't have to think. Whatever I was doing, I would have to stop it because I would go low. Yeah, I wouldn't have to think about that (12_15y_M).<br><br>It was quite a positive impact just in terms of, especially in group things or like supervisions, that type of thing when it's not really a good time to have to stop and give yourself insulin or like deal with something because you know it's already doing it for you. So I think it definitely like saved probably time like in those situations, but also just like having to have a conversation about it and then go and do something and it's like the extra… It's saving you from having to like exit and go and check things outside and come back. And so I think it was quite good for that as well because I knew it was just doing it in the background... Yeah, I'm not sure how much impact on productivity really, but I guess it is once again the focus is uninterrupted more. So it improved in the same kind of way (04_20y_F).
Social life	Less worry over hypoglycaemia<br>I think it like it made things easier and as well. It would like if you've got less hypos, it's obviously you can go and do more (04_20y_F).<br><br>Going out was easier, but that was just really to uni because I haven't really gone out with friends often since lockdown and COVID. We don't really go out much anymore. But it was going to uni's been easier I guess because I don't have to be worried as much about lows while going outside or on the train or it was just more convenient and easier. I do not have to worry about having lows outside as often (06_18y_M).<br><br>Less need to use phone or pump<br>Before I really wanted to go out more but I was embarrassed to bolus and I'd have to say I'm gonna quickly go somewhere to bolus but (with the fully closed-

	loop) I didn’t have to do that. So it positively affected my social life (12_15y_M).<br><br>(Regarding usual care) My school doesn't allow phone. So when I take out my phone like everyone looks at me and stuff (11_14y_M).<br><br><u>More present in daily life due to less preoccupation with diabetes</u><br>I just went out more. It was a much happier time of life. I guess there was less worry. You didn't have to think about it. You weren't consciously worrying about it throughout the day (01_20_M).<br><br>(When talking about going on holiday to Italy with his girlfriend while using the closed-loop:) But throughout that time, it was kind of just clean sailing. I just didn't have to look down at it at all times. I had to do a set change but that's, again, just muscle memory at this point. So it was very good. It was very nice to use… I've been on holidays a few times when the entire time was just the heat change or the atmospheric change or the weather. The difference in weather can affect my sugars quite badly, and it's kind of spent the entire holiday just worrying about that or trying to fix it or trying to change it, whereas this time I just didn't have to touch it at all. (01_20y_M).<br><br>I think I've always kind of done everything I wanted to anyway, so it wasn't like a massive change but it definitely I think made things just easier still… Like if you go on a night out or something, then it's, you know you don't want to be thinking about your blood sugar or checking your levels. And it makes it definitely a lot easier to manage I think (04_20y_F).

Supplementary Table 5. Theme: Trial close-out.

Sub-theme	Participant quotations
Reflections on the fully closed-loop system	<u>Additional strengths of the system</u><br><u>*Boost*</u><br>I've eaten something without checking how much sugar it has and, oh, no, it's got a lot. So I'm going to Boost it just to try help pre-emptively, kind of, instead of the system reacting to my sugars, I know my sugars are going to rise, so I'm going to pre-emptively Boost it to keep it somewhat in level. And that did help. That did work quite well (01_20_M).<br><br>I mostly used Boost. When my blood sugar's like 14. Like if my sugar is not going down… Like after I go out like after we eat out. Or evening mostly night-time (11_14y_M).<br><br><u>*Ease-off*</u>

	Ease-off more if I noticed like if I was already fairly low and then say I was gonna do sport for two hours and kind of then just so there was the advance kind of preparation (04_20y_F).<br><br>Just before I will do PE I would ease off just to make sure I don't go low and my mom will tell me to do that (12_15y_M).<br><br>Limitations of the system<br>*Algorithm's slowness at managing high glucose levels*<br>Yeah, I guess it wouldn't be a dislike. I just think it's just something you'd have to get over. But how long it takes for the system to get used to you as in for the blood levels to go back to the normal states and things like that (06_18y_M).<br><br>I don't think like the time lag sometimes between like correcting things just with like when I'd end up being high for ages because it was going back and forth between more and less insulin and then it was going back up and I did feel like it was a bit prolonged sometimes and I did think sometimes, if I just injected myself or been on the Omnipod or whatever, I could have probably dealt with it quicker… So I guess particularly what the way the app dealt with the hyperglycaemia wasn't as great… (04_20y_F).<br><br>*Urgent low alarm*<br>The only thing I didn't like was with the notifications. You couldn't switch off the urgent low alarm, which I found to be quite a problem in like lectures or libraries or areas where you can't really have things going off… I think it would be great if there was more flexibility with that one (04_20y_F).<br><br>*Small insulin cartridge*<br>Given that the insulin also runs out quickly in that pump. So I was a bit wary about what food I was eating… This is a bit hard because as it is a small pump, you can't really fit much (02_14y_F).<br><br>I think it holds slightly less insulin. It meant I had to replace that more frequently, but that was probably the only kind of bigger downside apart from the tubing (04_20y_F).<br><br>*Tubing of pump*<br>I don't know how I felt about the wire because it would get in the way if I was running like at school doing PE it would like where you click like clipped it onto your waistband, it would fall off. Like the wire was annoying (05_13y_F).<br><br>The only thing I didn't like about the pump is the wire 'cause it was kinda annoying. Like it going on like the doorknobs as I went past. It gets stuck, it gets trapped something like that (11_14y_M).

Readjusting to standard insulin pump therapy	I mean a couple of times I had just started eating without carbing, so only took me like couple days (05_13y_F).<br><br>It took a week, a week or so to get used to having to put in the carbs again. But after that, it feels like I've gone back to normal (10_18y_M).<br><br>I became a lot worse at controlling my diabetes after I got off the closed loop because the things I forgot to worry about, I have to now worry about and the insulin and whatever I needed to give I now need to give again. So I guess I got worse. Since I'm on hybrid closed loop, it's not as bad (06_18y_M).<br><br>“It was really strange at first right having to actually remember when I was eating again to give insulin and using all the temporary basals again. Yeah, I mean, I just did go back to it again, but it was like you suddenly had a lot more to think about as well and it was right before I went into exams and stuff. So, then I was trying to remember that on top. So, it's definitely a bit more stressful“ (04_20y_F).<br><br>Practically not as much, but mentally it was a lot more. Yeah. And then I think that on top of that I'd just started third year as well. So, thinking about dissertation and uni and work and learn it kind of all went out the window for the first few weeks and then I eventually got homed back in I guess… Hypers and hypos weren't necessarily the worry. I guess with the actual diabetic and controlling the sugars wise it wasn't too bad. There were some really bad ones where it did go really high. Really low. But I think it was more mentally taxing of relearning it at this point (01_20_M).
Who would most benefit	I think younger kids as well, though 'cause it's easier for them to manage then isn't it? But if I had, if I had a younger child with it and then you know, send them to school and they're eating what they like and not having to worry about it too much would be a big, big strain off of a parent (parent of 08_14y_F).<br><br>I suppose probably people who are newly diagnosed, because I think it is a lot simpler. And so if you've not been exposed to all the methods of management it is going to be a lot harder to pick them up… But I think if I'd been struggling more, especially when I was younger, it would have had an even bigger kind of impact. Um, like when I didn't know as much about it or how to manage it myself. Uh, but yeah, think like young people in like the education system and stuff (04_20y_F).<br><br>People in high stress, not a lot of time. That's pretty much it. People that are either literally have the chance to glance at the phone or try sort it out within 30 seconds. Also those that, like me. Good example. Just going straight through into uni, where you're thrown to the other side of country, away

	from home, where everything's new, you don't really know what you're doing. You're still trying to put it all together. You’re not going to have as much time to kind of sort out your diabetes, I guess (01_20_M).

Supplementary Table 6. Free-text results from the Closed-loop Experience Questionnaire (n=22).

Q7. What did you like about the closed-loop system?

- I liked how I was able to feel normal again. No carb counting. No finger-pricking.
- Not having to worry about carbing for the food I'm eating. Less nagging about carbing.
- Not having to bolus/carb count. Less worry about preventing lows.
- Very easy
- I didn't have to worry as much about my levels
- Being able to eat with my family.
- Everything fully automated. Very useful in day-night lifestyle. Essentially (apart from insulin set insertions) haven't felt diabetic for 6 weeks.
- Did not have to do much with it.
- The fact I didn't have to worry about bolusing.
- Not have to do anything.
- it takes less time
- I did not have to look after and worry about my blood level as much. My HbA1c lowered.
- The system correcting my blood sugar level without me having to intervene.
- I didn't have to worry as much.
- I liked it all.
- I liked that I didn't have to worry about my glucose going high as much.
- That I had to less work
- That I don't need to do carb counting
- Done barely any work
- Self-regulation of my blood glucose. It managed to effectively sense when I was doing an activity that affect my diabetes and it responded accordingly.
- It felt like I didn't need to worry about my blood sugar levels as they were being continuously monitored and corrected. It was easy to use/store and it is easier than I expected to have a wire.

Q8. What are the things you did not like about the system?

- Nothing really.
- Nothing I liked it all
- Nothing (3)
- The wires. The algorithm adjusting to what insulin I needed.
- No way to switch off the urgent low alarm. A bit slow to react and fast to stop insulin delivery so prolonged highs.
- Better on iPhone
- The pump or the sensor. The pump had some annoying alarms and didn't have enough space for insulin so would have to change sets more. The sensor had connectivity and was paused. It also didn't stop me from going extremely high.
- How often I have to refill insulin.
- A few disconnection issues, phone range to pump and sensor. Pump had a few funny moments when changing battery whereby it fully suspended delivery + refused to reconnect to phone.
- Always seemed high and had to correct/boost often. Did not work quick enough.
- Trusting the system to bring my BG down when I see it going up.
- The tube.
- The cannula gave me 1-2 problems.

- After meals my blood level was high for prolonged times (4-5 hours)
- The system was alright, however, I disliked the tubing and having to clip a device to my trousers.
- The errors
- Started vibrating in school and it slightly embarrassed me
- Often mechanical/electrical faults to do with battery. A bit confusion and takes time to get used to it.
- It makes some activities (rugby, swimming) more difficult. Worried about being connected to a wire through the night. The cannula was quite thick, it didn't hurt but left a mark. The adhesive could be a bit more sticky, came lose quite easily.

Q9. Would you like the closed-loop system to have additional features? If yes, which?

- No (7)
- I thought the features accessible were good.
- Set change tracker incorporated so don't have to record separately as insulin refill wasn't matched up with this.
- No wire
- I don’t think so
- An option to turn off alarms.
- Have a backup sensor within pump to scan and auto-connect to phone instead of Bluetooth. It can take very long to reconnect between phone and pump (aka instant reconnect fail-safe). May sound silly (but it's the only thing I've wanted since I've had the pump)... can you add birthday + celebratory messages to flash on the pump) Have alternative set insertions (vertical needle + angled needle).
- A button to tell it when I have eaten, so it knows to give me more insulin.
- Not completed.
- Omnipod because it has no tube.
- No, it is quite effective the way it is.
- An easier scroll menu
- The pump to be wireless
- Nothing
- Stronger adhesive. Possibly no wire.
